# The genome landscape of Hong Kong feral cattle as a unique genetic resource

**DOI:** 10.1016/j.isci.2026.115465

**Published:** 2026-03-24

**Authors:** Xiaoyu Luo, Xiaoran Lu, Yan Ren, Yifan Cao, Xuewei Liu, Mario Barbato, Paolo Ajmone-Marsan, John L. Williams, Rick Tearle, Richard A.L. Brown, Michael P. Reichel, Chuzhao Lei, Ningbo Chen, Wai Yee Low

**Affiliations:** 1 Key Laboratory of Animal Genetics, Breeding and Reproduction of Shaanxi Province, College of Animal Science and Technology, Northwest A&F University, Yangling 712100, China; 2 Davies Livestock Research Centre, School of Animal and Veterinary Sciences, The University of Adelaide, Roseworthy, SA 5371, Australia; 3 Department of Veterinary Sciences, Università degli Studi di Messina, 98168 Messina, Italy; 4 Department of Animal Science, Food and Technology–DIANA, Università Cattolica del Sacro Cuore, 29122 Piacenza, Italy; 5 College of Veterinary Medicine and Life Sciences, City University of Hong Kong, Tat Chee Avenue, Kowloon Tong, Hong Kong 999077, China; 6 Department of Population Medicine and Diagnostic Sciences, Cornell University College of Veterinary Medicine, Ithaca, NY 14853, USA

**Keywords:** Phylogenetics, Genomic analysis, Genomics

## Abstract

In the Hong Kong Special Administrative Region of China, there is a feral cattle population that has not been well characterized genetically. In this study, we used high-coverage (∼30×) whole-genome sequencing from 30 Hong Kong feral (HKF) cattle and compared them to 116 individuals from four representative populations worldwide. Our analyses revealed that the HKF cattle have high genetic diversity in the face of a declining effective population size, suggesting their substantial and yet untapped genetic potential. We also identified introgression events that occurred prior to the divergence between HKF cattle and other East Asian indicine populations, which shaped the adaptation of HKF cattle in Asian agro-ecologies. Moreover, we identified positive selection in HKF cattle for environmental adaptation, particularly in traits related to heat tolerance, bone strength, and coat color. Our findings provide insights into the genetic origin and unique adaptation of HKF cattle.

## Highlights

• HKF cattle represent a unique gene pool of indigenous East Asian populations
• HKF cattle harbor substantial yet untapped genetic potential
• Introgression shaped HKF cattle genomes before splitting from other East Asian populations
• Selected genomic loci associated with environmental adaptation were found in HKF cattle

## Graphical abstract

**Figure:**
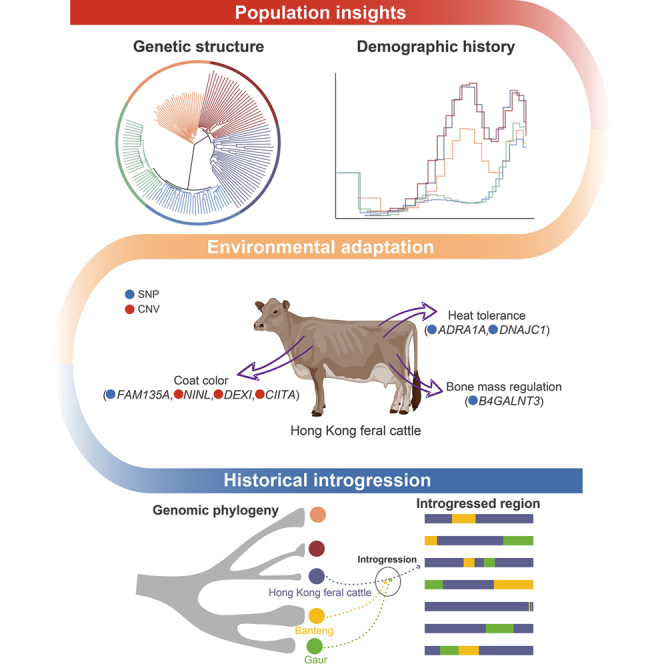

## Introduction

Cattle are one of the main livestock species worldwide and have played an important role in agriculture and society since their domestication. They were introduced into new regions through human migrations, where exposure to novel ecological conditions imposed strong selective pressures on their genomes over a brief period.^1^ The evolutionary consequences of these pressures were further shaped by human management. Populations under intensive management often exhibited restricted genetic and phenotypic diversity, whereas unmanaged populations typically retained higher diversity and were better adapted to local environments.^2^

In the Hong Kong Special Administrative Region of China, there are about 1,000 feral cattle^3^ that inhabit urban environments, which represents a unique context for feral populations worldwide, but little is known about their genetic background and adaptive changes. Previous studies indicated that the early geographical history of Hong Kong region was attributed to the Lingnan region in southern China.^4^ In this region, cattle likely arrived by a coastal route during the eastward spread of domesticated indicine cattle.^5^ Subsequent admixture with local wild or domesticated banteng and gaur led to increased genetic diversity.^5^^,^^6^^,^^7^ Due to the effects of geographical isolation, the Hong Kong cattle population may be distinct from mainland populations. In the 1970s, rapid urbanization and economic development in Hong Kong led to the abandonment of cattle farming, and as a result, the Hong Kong cattle started to roam freely and are now considered a feral population. They are therefore exposed to disease, have free mate choice, and other pressures associated with surviving in natural and sometimes urban environments. Interestingly, despite being a small population, the Hong Kong feral (HKF) cattle displayed high phenotypic diversity in terms of horn shape and orientation, coat color, hump size and shape, tail length, and conformation. Hence, the HKF population offers a unique opportunity to study the effects on the genome and to characterize the needs and diversity of this unique population.

One previous study based on single-nucleotide polymorphism (SNP) array data showed no evidence of gene flow from any of the Asian local breeds to the HKF cattle.^8^ Meanwhile, a study based on Y chromosome and mitochondrial DNA data reported the pattern of introgression of the indicine genome across southern China.^5^ Notably, both studies support the introgression from wild bovine species into the genome of Asian and HKF cattle. The findings of these studies indicate that the evolutionary history of HKF cattle has been significantly influenced by introgression. Furthermore, due to biased sampling, different methodological perspectives, and limited genetic material, previous studies have not examined the genetic history and adaptive evolution of HKF cattle. In this study, by analyzing whole-genome sequencing (WGS) data of 30 HKF cattle, we revealed the genetic origin, introgression from wild species, adaptive evolution, and the genetic basis underlying the phenotypic characteristics of HKF cattle. Our findings provide insights into the genetic history of HKF cattle and enhance our understanding of adaptive evolution in feral cattle.

## Results

### Genome sequencing and SNP variant calling

**Figure 1:**
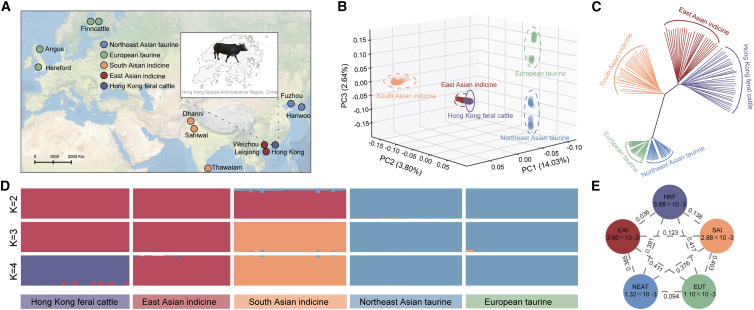
Population structure and relationships of HKF cattle to other cattle populations (A) Geographic map of sample locations and other cattle populations. (B) PCA of selected cattle populations. (C) NJ tree of the relationships between the cattle populations. (D) The ancestral components inferred using ADMIXTURE for *K* from 2 to 4. (E) Genetic diversity and population differentiation across five populations. The values inside the circles represent nucleotide diversity, while the values on the connecting lines between populations indicate the population *F*_ST_.

Individual genomes of 30 HKF cattle were sequenced to an average 29.89× coverage (Table S1). Genotypes for these were jointly called with publicly available genomes of four cattle populations, including European taurine (Hereford, Angus, and Finncattle), Northeast Asian taurine (Hanwoo and Fuzhou), East Asian indicine (EAI) (Weizhou and Leiqong), and South Asian indicine (SAI) (Sahiwal, Thawalam, and Dhanni) (Figure 1A). In total, all 146 samples were aligned to the *Bos taurus* reference assembly ARS-UCD1.2 with an average mapping rate of 99.50%. Variants were called and filtered using a customized pipeline (see the STAR Methods section), resulting in the retention of ∼57.06 million bi-allelic SNPs. Functional annotation revealed that the majority of SNPs were located in intergenic (58.59%) and intronic regions (38.23%), while the rest were in the regions upstream and downstream (1.25%) of open reading frames and untranslated regions (1.01%). Exons contained 0.92% of the SNPs, with 496,136 synonymous SNPs and 11,957 nonsynonymous SNPs (Table S2; Figure S1).

In the HKF cattle population, we compared the SNPs detected between WGS and SNP50k array data.^8^ After pruning for missing data, we retained 33,720 SNPs in the array, of which 16,334 were monomorphic whereas 17,386 are low-frequency variants (minor allele frequency [MAF] ≤ 0.05) (Table S3). In contrast, WGS captured genetic variation comprehensively across the allele frequency spectrum. For comparison with the WGS data, 30,813 variants could be lifted over to ARS-UCD1.2 coordinates. A total of 15,614 SNPs were common to both datasets. We then calculated heterozygosity (*Ho*) and inbreeding coefficient (*F*) for the WGS data and compared them with the published full SNP panel (*Ho* = 0.144 ± 0.01, *F* = 0.57 ± 0.03) and ancestral SNP panel (*Ho* = 0.268 ± 0.02, *F* = 0.35 ± 0.05).^2^ Our WGS data showed higher heterozygosity (*Ho* = 0.288 ± 0.02) and lower inbreeding coefficient (*F* = 0.25 ± 0.05).

### Genetic structure and differentiation

To explore the genetic relationships among HKF and other cattle populations, we performed principal-component analysis (PCA), neighbor-joining (NJ) tree, and ADMIXTURE analyses of the autosomal SNP genotype data. Principal component (PC)1 explained 14.03% of total variance and distinguished *B. taurus* from *B. indicus*, while the second and third PCs, which accounted for 2.64% and 3.80% of total variance, clustered HKF with EAI and distinguished HKF from SAI (Figures 1B, S2A, and S2B). Similar results were observed in the NJ tree (Figure 1C). To further investigate the ancestry of the populations, we used ADMIXTURE to calculate delta *K* for each *K* from 1 to 4, where *K* represents the assumed number of ancestral populations. *K* = 2 divided *B. taurus* and *B. indicus* ancestry; when *K* = 3, HKF shared genome ancestry with EAI; and when *K* = 4, HKF showed distinct genetic patterns compared to the other four populations (Figure 1D). In addition to autosomal analysis, we examined the Y chromosome to investigate the male histories of HKF cattle. The Y3 haplogroup belongs to *B. indicus*, and the HKF populations shared the Y3A sub-haplogroup with the EAI populations, whereas the sub-haplogroup Y3B was predominantly found in SAI (Figure S3). Consistent with SNP array results showing HKF as a distinct cluster with highly divergent SAI ancestry, our WGS analysis confirmed its close genetic relationship with EAI ancestry.

Fixation index (*F*_ST_) also showed that HKF cattle had the greatest similarity to EAI compared with the other populations (Figure S4). We calculated nucleotide diversity (π) of the populations to measure the degree of polymorphism (Figure 1E). HKF cattle showed the highest level of nucleotide diversity, followed closely by EAI.

### Inbreeding and demographic history of HKF cattle

**Figure 2:**
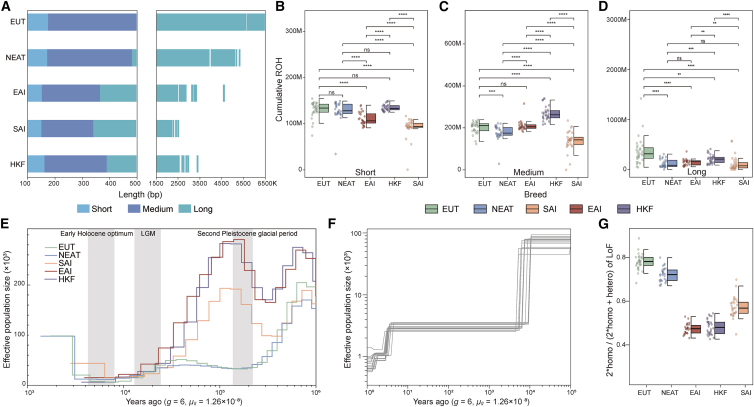
Characterization of inbreeding and demographic history in the HKF population (A–D) Distribution of ROH segments ordered and colored by size class in all populations. Cumulative size of short (B), medium (C), and long ROH (D) segments across all cattle populations based on the Gaussian mixture model. Differences between populations were tested by Wilcoxon rank-sum tests. Asterisks denote ∗*p* < 0.05, ∗∗*p* < 0.01, ∗∗∗*p* < 0.001, and ∗∗∗∗*p* < 0.0001. (E) Demographic history inferred from the MSMC2 method. The large gray-shaded boxes illustrate the early Holocene Optimum, the last glacial maximum (LGM), and the second Pleistocene glacial period. (F) Effective population sizes for the HKF population were inferred using SMC++,^15^ computed on 15 groups of 10 randomly selected samples. (G) The ratio of homozygous LoF alleles in all populations.

Inbreeding in HKF populations was evaluated by screening runs of homozygosity (ROH) across the genome. In terms of population structure, short, medium, and long ROH reflect ancient haplotypes predating continental migration, background relatedness, and recent parental relatedness, respectively.^9^^,^^10^ Among the three populations with indicine ancestry (EAI, SAI, and HKF), the HKF cattle had the most ROH, regardless of the length of ROH analyzed (Figures 2A–2D). The European taurine (EUT), which has experienced reproductive barriers and intensive artificial selection, had the highest number of cumulative long ROH among all five populations (Figure 2D). The number of long ROH in HKF was second only to the inbred EUT, indicating that HKF has a greater inbreeding risk than other indicine populations. Analysis of long ROH specific to HKF revealed a total of 270 genes, including a region on BTA13 (22.66–23.16 Mb) harboring genes associated with energy balance (*SKIDA1*^11^), growth traits (*MLLT10*^12^), and heat resistance (*DNAJC1*^13^^,^^14^). Haplotype analysis revealed distinct patterns relative to other groups, indicating potential genetic differentiation and selective pressure in HKF cattle (Figure S5). Cumulative ROH length was highly correlated with ROH number in all three ROH length classes (short, medium, and long; R > 0.9; *p* < 0.01) (Figure S6).

The HKF had the highest medium ROH among the populations studied (Figure 2C), which suggests that its effective population size is small. To further investigate the changes in effective population size over time in the HKF population, we estimated the historical Ne using the multiple sequentially Markovian coalescent (MSMC2) method,^16^ which revealed that the HKF experienced a great population decline at 20–30 kya (Figure 2E). The population size history of HKF was similar to that of EAI but differed from that of the taurine populations. The recent effective population size of HKF was estimated using SMC++,^15^ which revealed that HKF Ne was lowest 7–9 kya, after which the population size remained constant (Figure 2F).

As the HKF cattle have a high level of inbreeding, loss-of-function (LoF) mutations may have accumulated at high frequency in this population, which is concerning for conservation genetics. The LoF mutations were screened across the entire genome of the five populations to investigate putatively derived deleterious mutations. The HKF cattle had a lower homozygous deleterious mutation load than SAI (Figure 2G).

### Historical introgression

**Figure 3:**
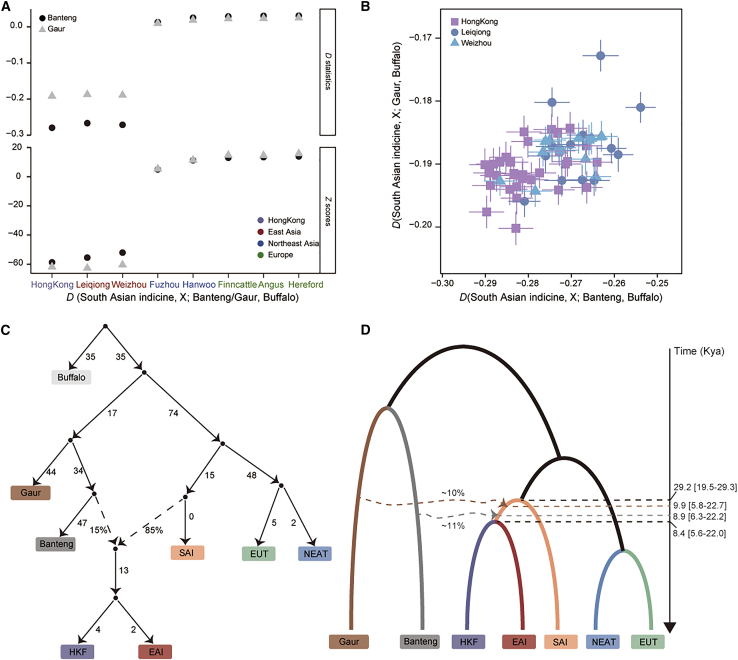
Introgression ratio and history of HKF cattle (A) *D* statistics with the form *D* (W, X; Y, Z) for banteng/gaur and five populations. (B) Pairwise *D* statistics assessing allele sharing between HKF cattle and banteng/gaur, and comparisons of allele sharing between EAI breed cattle and banteng/gaur. (C) Origin history with 1 admixture event inferred by qpgraph. (D) Demographic model evaluated by fastsimcoal. The 95% confidence intervals are shown in parentheses.

Previous studies have reported introgression from banteng and gaur into HKF cattle,^8^ and suggested admixture from banteng and gaur in EAI cattle.^5^ These findings indicated a significant contribution of wild cattle to the indicine populations in East Asia. In this study, SNPs derived from high-coverage WGS were used to calculate *D* statistics^17^ and RFMix^18^ to verify and quantify introgression from banteng and gaur into HKF cattle. The results showed that the proportions of banteng and gaur ancestries ranged from 10.74% to 11.81% and from 9.70% to 11.08%, respectively, in the HKF genomes. The proportions of banteng and gaur ancestries in the HKF genome were greater than those in the EAI genome (Figures 3A and 3B; Tables S4 and S5). We further explored the historical gene flow and introgression events using qpgraph and TreeMix.^17^^,^^19^ When testing without admixture, the topologies based on qpgraph and TreeMix were consistent with the topology of the NJ tree (Figures S7 and S8), showing that the common ancestors of HKF and EAI cattle diverged from SAI cattle. When modeling one admixture event, qpgraph and TreeMix analysis indicated an introgression from banteng and gaur into the common ancestor of HKF and EAI cattle (Figures 3C and S8). We therefore explored the possible introgression times from banteng and gaur into HKF cattle using fastsimcoal.^20^ The estimated banteng introgression occurred ∼8.9 kya (95% confidence interval [CI]: 6.3–22.2 kya) with an admixture fraction of ∼10% in HKF cattle, while gaur introgression was estimated to have occurred ∼9.9 kya (95% CI: 5.8–22.7 kya) with an admixture fraction of ∼9% (Figure 3D). This is consistent with the qpgraph, suggesting that banteng and gaur introgression occurred prior to the divergence of HKF from EAI cattle.

### Effects of introgression on adaptation

To evaluate the effects of introgression on adaptation of HKF cattle, we used the *U50* statistic to detect adaptive introgressed genes. We found 1,403 genes in the HKF genomes to be of banteng origin and 1,553 genes to be of gaur origin, with 1,234 genes shared by both banteng and gaur (Figures S9A and S9B). These overlapping genes were highly enriched in Kyoto Encyclopedia of Genes and Genomes (KEGG) and Gene Ontology (GO) pathways (corrected *p* value <0.05) of oxygen binding, transport and carrier activity (*HBM*, *MB*, *HBQ1*, *HBA*, *HBZ*, and *HBA1*), and bitter taste receptor activity (*TAS2R7*, *TAS2R16*, *T2R12*, *TAS2R8*, and *TAS2R38*) (Table S6). In addition, we observed that the most introgressed region on BTA9 (*FAM135A*) harbored genes relevant to coat color,^21^ lipase activity, and intramuscular fat deposition.^22^^,^^23^ The geographic distribution of haplotypes in global cattle populations revealed that the introgressed gaur and banteng haplotypes had the highest frequency in HKF and EAI cattle. This finding was further supported by the *F*_ST_ analysis (Figure S9C), phylogenetic analysis (Figure S9E), and the extent to which the haplotypes were shared across populations (Figure S9D).

### Adaptive selection of HKF cattle to environmental stresses

**Figure 4:**
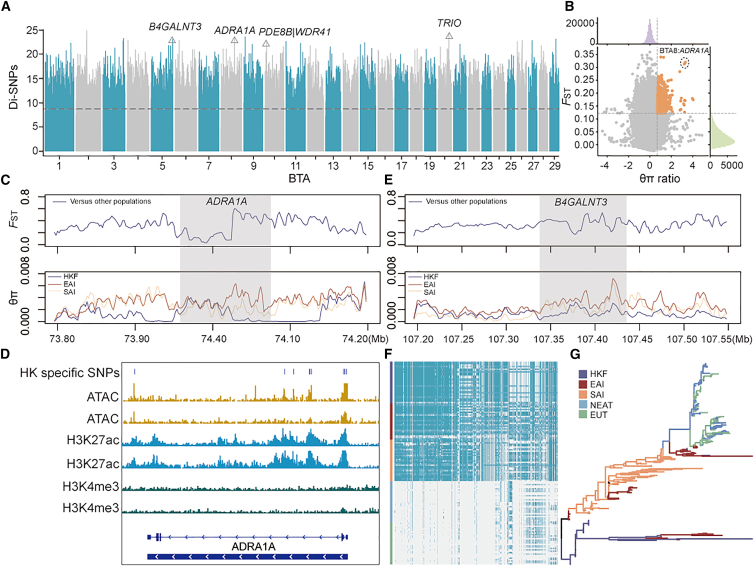
Candidate selection signature variants in HKF cattle (A) Di-HKF are the 0.1% most significant Di-SNPs identified when comparing HKF versus other populations. (B) Distribution of the *F*_ST_ (*x* axis) and *θ*π ratio (*y* axis) between HKF and EAI cattle based on 50 kb windows with 20 kb steps. The loci with the highest values are highlighted by a dashed circle on BTA8. (C) The *F*_ST_ and *θπ* estimates for SNPs in the selected region on BTA8 between HKF and other indicine cattle based on 5 kb windows with 2 kb steps. (D) Overlap of HKF SNPs in *ADRA1A* with open chromatin, enhancer, and promoter markers. (E) The *F*_ST_ and *θ*π estimates for SNPs in *B4GALNT3* gene on BTA5 between HKF and other indicine cattle based on 5 kb windows with 2 kb steps. (F) SNPs with MAF >0.05 were used to construct haplotype patterns. The major allele at each SNP position in HKF cattle is colored magenta. (G) Haplotypes of the relationships among all populations on the *B4GALNT3* genes. Phylogenetic tree constructed from SNPs in *B4GALNT3* genes.

Our genome-wide analysis showed that HKF cattle exhibit a genetic pattern that is distinct from other cattle. To identify signatures of positive selection in the feral population, we compared the genomes of HKF cattle with other populations of cattle using the Di statistic.^24^ A total of 653,861 SNPs (0.1%) had significant allele frequency differences, which may be targets of local adaptation (Figure 4A). Among these, strong signals of differentiation were obtained in the regions containing genes related to neurological development (*TRIO*),^25^ thyroid development (*PDE8B*),^26^ and autoimmune regulation (*WDR41*).^27^ A significant selection signature was observed for *ADRA1A* gene on BTA8 (Figure S10), which is associated with efficient recovery from heat stress.^28^ The *ADRA1A* gene had the strongest differentiation between HKF and EAI cattle for the *F*_ST_ and *θπ* ratios (Figure 4B). Evidence of selection on this gene in HKF cattle was provided by high *F*_ST_ values and low genetic diversity (Figure 4C). In addition, we detected 12 Di-SNPs in the *ADRA1A* gene, which are putative functional polymorphisms (Figure 4D; Table S7). These variants affect the enhancer and promoter activity of *ADRA1A*, thereby potentially influencing its expression. Notably, the SNP rs517553622 (C > T) located in the promoter of *ADRA1A* was predicted to enhance the binding of KLF17 transcription factor, which is involved in the regulation of immune responses (Table S8).^29^ Another strong selection signature was located on BTA5, covering the *B4GALNT3* gene, which contributes to bone health and supports survival in the wild (Figure 4A).^30^ It had high *F*_ST_ between HKF and other indicine cattle, as well as a likely recent selection signal in HKF cattle seen from the *θπ* analysis (Figure 4E). The haplotypes of the *B4GALNT3* gene in HKF cattle are distinct from other cattle populations (Figures 4F and 4G).

### Copy number variation calling and identification of copy number variation regions

**Figure 5:**
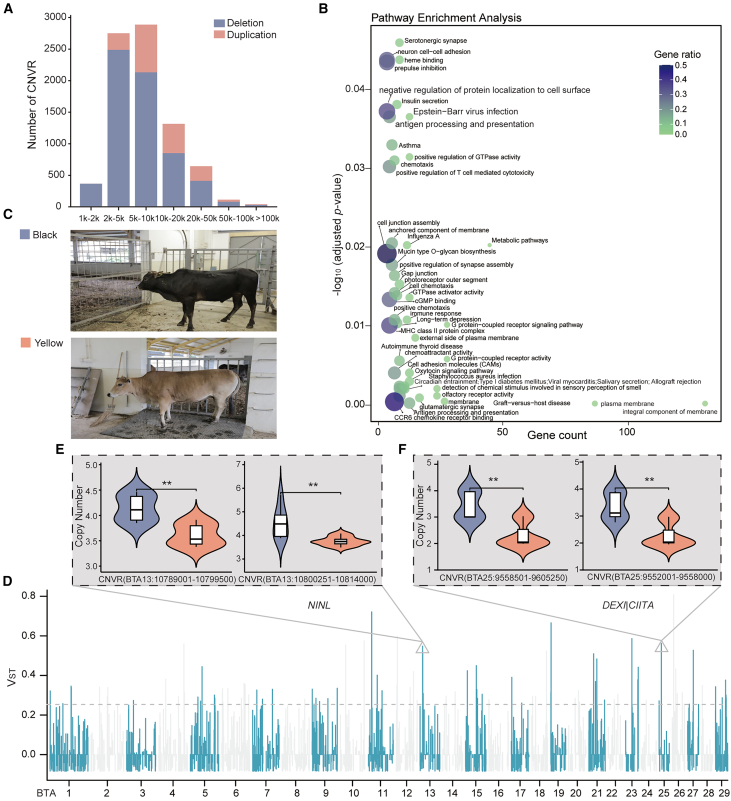
Detection and functional annotation of CNVRs (A) Counts of each CNVR type in HKF cattle, with purple and pink bars representing deletion and duplication, respectively. (B) Enrichment analysis based on the CNVR-related genes. (C) Photographs of example HKF cattle used in this study, showing the black and yellow color phenotypes (adapted from Barbato et al.^8^). (D) Manhattan plot of V_ST_ statistics between black and yellow color phenotypes in HKF cattle. (E) Estimated copy numbers of CNVRs on BTA13 (CNVR-*NINL*) between yellow and black color phenotypes. (F) Estimated copy numbers of CNVRs on BTA15 (CNVR-*DEXI* and CNVR-*CIITA*) between yellow and black color phenotypes.

Our mapped sequence data had an average depth of 29.89× per sample, which was sufficient for detecting copy number variations (CNVs) based on read depth.^31^ After CNV calling and filtering, a total of 8,120 CNV regions (CNVRs) were identified on autosomes (Table S9), which consisted of 6,371 deletions and 1,749 duplications compared with the ARS-UCD 1.2 reference genome (Figure 5A). KEGG and GO analyses revealed enrichment of genes involved in biological processes relevant to olfactory receptor activity, the immune system, and response to autoimmune thyroid processes (corrected *p* value <0.05) (Figure 5B; Table S10). Among these, a set of genes associated with the immune response, including *BLA-DQB*, *TNFSF10*, *RAET1G*, *BOLA-DQB*, *CD1A*, *MIC1*, *IL32*, *BOLA-DQA5*, *BOLA-DQA2*, and *BOLA-NC1*, may affect the response of HKF cattle to parasitic pathogens.

To investigate the impact of candidate CNVRs on coat color of HKF cattle, we calculated the V_ST_ between individuals with black and yellow coats. A total of 143 CNVRs were found to have a V_ST_ value greater than or equal to the highest 1% of values (Figures 5C and 5D). Among these, a total of 51 genes were annotated as differentiated CNVRs. Four of these CNVRs were present in three genes that were associated with coat color (*NINL*, *DEXI*, and *CIITA*). We compared the copy numbers of overlapping CNVRs for black and yellow HKF cattle individuals. In black individuals, CNVR-*NINL*, CNVR-*DEXI*, and CNVR-*CIITA* had higher copy numbers than in yellow individuals (Figures 5E and 5F). We hypothesize that these genes may play important roles in the coat color phenotype of HKF cattle.

## Discussion

HKF cattle exist primarily as feral animals in Hong Kong, China, and the population size has been declining.^32^ Our study used high-depth WGS data to investigate the genetic structure, demographic history, introgression time frames, and genetic architecture of adaptive traits of HKF cattle. The use of WGS data is an advantage over our previous work on HKF cattle that relied on BovineSNP50 SNP chip^8^ because it reduces ascertainment bias. The BovineSNP50 chip^33^^,^^34^ is designed based on *B. taurus* and, as such, is not representative of the genetic diversity of HKF cattle that have substantial indicine genetics. Moreover, our dataset allowed for the detection of CNVs that were missed in previous studies relying primarily on genotyping arrays.^8^

Here, we showed that the ancestral contributions of HKF cattle came from EAI cattle, a pattern that was supported by the distribution of Y chromosome uniparental lineages. We also found that the HKF cattle exhibit distinct genetic patterns and internal uniformity, likely shaped by prolonged geographic isolation. We quantified inbreeding in HKF cattle by identifying ROH. Our analysis revealed that ROH length and number in HKF cattle were significantly higher than in other indicine cattle. The high degree of inbreeding in HKF cattle may be partly explained by limited gene flow with mainland populations during Hong Kong’s 151 years as a British colony (1842–1941 and 1945–1997). Interestingly, we observed fewer homozygous mutations that were predicted to be deleterious (LoF variants) in HKF cattle compared to the other cattle groups included in this study. Many predicted deleterious alleles were found in the heterozygous state. When combined with the small effective population size of HKF cattle, they pose a significant threat to future population’s viability. It is possible that inbreeding in HKF cattle has resulted in the purging of deleterious alleles through purifying selection. Moreover, associative overdominance, whereby heterozygous loci mask deleterious effects, may also contribute to this pattern. Both mechanisms warrant further investigation to clarify their roles.

We found evidence of adaptive introgression of banteng and gaur ancestry in HKF cattle, consistent with EAI cattle.^5^ The banteng and gaur introgression occurred before the divergence between HKF and EAI cattle. The high proportion of the banteng/gaur ancestry in these populations may be due to their geographic location, which served as the primary entry point of indicine cattle into East Asia.^5^ The banteng and gaur genes introgressed into HKF were enriched in biological pathways of oxygen binding, transport and carrier activity, and bitter taste receptor activity. In particular, the bitter taste receptor activity pathway included several target genes (*TAS2R7*, *TAS2R16*, *T2R12*, *TAS2R8*, and *TAS2R38*), which are associated with local adaptation to different food sources. Preference for different food sources due to taste is considered a strong driving force in ecological speciation.^35^^,^^36^^,^^37^ The oxygen binding pathway has been shown to be associated with disease and parasite tolerance in EAI cattle.^5^^,^^38^ Generally, energy homeostasis and color variation can influence the adaptability and survival of species. In the HKF population, *FAM135A*, that is introgressed from banteng, was found to significantly influence coat color,^21^ potentially contributing to the adaptation of HKF cattle to tropical and subtropical environments. *FAM135A* has been found to be associated with the lipid metabolism pathway and maintenance of homeostasis.^22^^,^^23^

Genomic adaptations to diverse environments are increasingly recognized as vital for conservation and food security, with rapid evolution shaping population productivity and viability in the face of climate change.^39^ Our analyses identified unique and clear signals of recent selection in HKF cattle, which reflect adaptation to a free-ranging environment. One of these regions contained the gene *ADRA1A*, which is associated with heat stress recovery.^40^ *ADRA1A* is a member of the G protein-coupled receptor superfamily that enhances adipocyte thermogenesis^41^ and changes feeding behavior due to heat stress.^42^ Variants in *ADRA1A* identified in this study revealed SNPs in predicted promoter and enhancer regions from assay for transposase-accessible chromatin using sequencing (ATAC-seq) and chromatin immunoprecipitation sequencing (ChIP-seq) data. Notably, the SNP in the promoter was predicted to enhance the binding of KLF17, a transcription factor involved in immune regulation.^29^ These non-coding variants may play a role in regulating its expression and facilitating the adaptation of HKF cattle to hot tropical ecosystems in the absence of human heat-stress mitigation management. Although the open chromatin region data came from Hereford cattle, they provide a basis for the tentative identification of conserved regulatory regions.^43^ Further studies are needed to account for potential differences in HKF cattle for more accurate inference of regulatory regions. We also identified a selection signal for *B4GALNT3* located at BTA5 in HKF cattle, with a haplotype that was different from the other four populations analyzed. We observed a mutation in *B4GALNT3* that affects protein structure. *B4GALNT3* is associated with circulating sclerostin in humans,^44^ and it may increase bone mass, reducing fracture risk.^30^ The positive selection signals in the genes *ADRA1A* and *B4GALNT3* may reflect the adaptation of HKF cattle to heat stress and environmental stress.

CNVs can affect gene function by altering gene dosage and structure and thus may play a role in phenotypic evolution.^45^^,^^46^^,^^47^^,^^48^ Our analysis of the number of CNVs in HKF cattle revealed that compared to the reference genome, deletions significantly exceeded the expansions. Pathway annotation of the expanded CNVRs identified in HKF cattle revealed a significant enrichment of genes related to the immune system. This suggests that HKF cattle may possess genetic features related to adaptive immune function that are potentially associated with survival and reproduction in their specific environments. The genes *BOLA-DQB*, *BOLA-DQA5*, *BOLA-DQA2*, and *BOLA-NC1* located on BTA 23, which are part of the bovine major histocompatibility complex (MHC) class II gene family, are in regions under selection and play key roles in immune response and adaptation to tropical conditions.^49^ Importantly, HKF cattle have not been subjected to recent artificial selection for production and morphological traits, which has led to phenotypic diversity in populations. In this study, we observed that the *NINL* gene differed between the two coat colors of HKF cattle. *NINL* has been shown to be involved in microtubule organization, leading to the dispersion of lysosomes toward the cell periphery, thereby affecting the distribution of melanosomes.^50^^,^^51^ The expression of *NINL* is higher in Asian skin, which may regulate the distribution of melanosomes, contributing to the distinct skin color differences compared to Caucasians.^52^ We hypothesize that *NINL* may have a role in the coat color phenotypic variations seen in HKF cattle. *DEXI* and *CIITA* have been shown to be associated with black color in camels.^53^

In this study, we explored the unique interactions between the demography and genetics of HKF cattle to better understand the evolutionary history of this population. We provided evidence that the ancestral contributions of HKF cattle came from EAI cattle. We identified wild introgression into HKF cattle, likely a result of their geographic position as a gateway for indicine migration into East Asia. At the genomic level, we were able to find several genetic variations that may be adaptations to the environment, potentially related to heat tolerance, survival, and disease resistance. Practically, HKF cattle have developed into a genetically distinct population clearly differentiated from their domestic ancestors. These results indicate how animals free from human management have to reshape their genome to cope with a harsher environment as they are unprotected from biotic and abiotic stress. This adaptation is at the expense of diversity caused by purifying selection and increased inbreeding due to mating of relatives from families having higher fitness under natural conditions.

### Limitations of the study

This study provides insights into the genetic origin and unique adaptation of HKF cattle. One limitation lies in the lack of breed-specific open chromatin data, which restricts the direct assessment of selection on non-coding regulatory regions. Without such data, inferences about regulatory adaptation are indirect and may not fully reflect the breed’s unique regulatory landscape. Generating open chromatin datasets in relevant tissues would allow a more precise characterization of regulatory selection. Another limitation is that the functional effects of candidate loci, particularly those associated with coat color, remain untested. Targeted *in vivo* and *in vitro* studies will be necessary to validate these loci and test the hypotheses proposed here. Additionally, potential sex-specific effects cannot be excluded, and future studies should incorporate sex as an important variable.

## Resource availability

### Lead contact

Further information and requests for resources and reagents should be directed to and will be fulfilled by the lead contact, Wai Yee Low (wai.low@adelaide.edu.au).

### Materials availability

This study did not generate new unique reagents.

### Data and code availability

• The newly generated whole-genome sequence data from this study have been deposited in the National Genomics Data Center (NGDC), China National Center for Bioinformation, under the Bioproject accession number PRJCA040422. The details of aforementioned data and other downloaded publicly available data used in this study are provided in Table S1.
• All analysis code has been deposited in the GitHub repository and is publicly available as of the date of publication. Accession numbers are listed in the key resources table.
• Any additional information required to reanalyze the data reported in this paper is available from the lead contact upon request.

## Acknowledgments

This work was supported by National Key R&D Programs of China (grant no. 2021YFF1001000), the 10.13039/501100001809National Natural Science Foundation of China (32341054 and 32522096), and the China Agriculture Research System of MOF and MARA (CARS-37). W.Y.L., J.L.W., and R.T. were supported by the Davies Bequest through the Davies Livestock Research Center of the University of Adelaide. We thank the High-Performance Computing (HPC) of Northwest A&F University (NWAFU) for providing the computing resources. We gratefully acknowledge all participants who contributed to this study. The cow illustration used in the graphical abstract was provided by BioGDP.com (https://biogdp.com/).^54^

## Author contributions

W.Y.L. and N.C. designed and supervised the project. X. Luo and X. Lu performed the majority of analysis. Y.R., X. Liu, and Y.C. were involved in data visualization. X. Luo, N.C., Y.R., W.Y.L., and C.L. wrote, reviewed, and edited the manuscript with input from all authors. M.P.R. and R.A.L.B. collected the samples and revised the manuscript. J.L.W. initiated the study, coordinated sample collection, and revised the manuscript. P.A.-M., M.B., and R.T. contributed ideas for analysis and revised the manuscript.

## Declaration of interests

The authors declare no competing interests.

## STAR★Methods

### Key resources table

**Table:** 

REAGENT or RESOURCE	SOURCE	IDENTIFIER
**Biological samples**		

Hong Kong feral cattle (HKF)	Hong Kong Special Administrative Region of China	N/A

**Deposited data**		

Resequencing data of Hong Kong feral cattle (HKF)	This study; National Genomics Data Center (NGDC)	PRJCA040422
Resequencing data of East Asian indicine cattle (EAI)	National Center for Biotechnology Information search database (NCBI)	PRJNA658727; PRJNA379859; PRJNA283480
Resequencing data of South Asian indicine cattle (SAI)	National Center for Biotechnology Information search database (NCBI)	PRJNA658727; PRJNA379859
Resequencing data of European taurine cattle (EUT)	National Center for Biotechnology Information search database (NCBI); European Bioinformatics Institute (EBI)	PRJNA176557; PRJEB28185
Resequencing data of Northeast Asian taurine cattle (NEAT)	National Center for Biotechnology Information search database (NCBI)	PRJNA210519; PRJNA1085861
Resequencing data of Buffalo cattle	National Center for Biotechnology Information search database (NCBI)	PRJNA547460
Resequencing data of Banteng and Gaur	National Center for Biotechnology Information search database (NCBI); European Bioinformatics Institute (EBI)	PRJNA325061; PRJNA658727; PRJEB31621; PRJNA427536

**Software and algorithms**		

Burrow-Wheeler Aligner	https://github.com/lh3/bwa	v.0.7.13
SAMtools	https://github.com/samtools/	v.1.9
Genome Analysis Toolkit (GATK)	https://github.com/broadinstitute/gatk/releases	v.3.8.1.0
ANNOVAR	https://github.com/WGLab/doc-ANNOVAR	v2016-02-01
PLINK	https://www.cog-genomics.org/plink/	v.1.9
EIGENSOFT	https://github.com/argriffing/eigensoft	v4.2
ADMIXTURE	https://dalexander.github.io/admixture/	v1.3.0
MEGA	https://www.megasoftware.net/	v11.0
VCFtools	https://vcftools.github.io/index.html	v0.1.16
Mclust R package	https://mclust-org.github.io/mclust/	v6.1.1
BEDTools	https://github.com/arq5x/bedtools2	v.2.25.0
SnpEff	http://pcingola.github.io/SnpEff/	v4.3
MSMC2	https://github.com/stschiff/msmc2	V2.1.0
SMC++	https://github.com/popgenmethods/smcpp	v1.15.4
RepeatMasker	http://www.repeatmasker.org	v.4.0.5
Trim Galore	https://github.com/FelixKrueger/TrimGalore	v.0.6.10
Bowtie2	https://bowtie-bio.sourceforge.net/bowtie2	v.2.4.5
Picard	http://broadinstitute.github.io/picard	v.2.20.2
Macs2	https://github.com/macs3-project/MACS	v.2.2.7.1
MEME	https://github.com/cinquin/MEME	v5.5.2
RFMix	https://github.com/slowkoni/rfmix	v2.02
ADMIXTOOLS	https://github.com/DReichLab/AdmixTools	v7.0.2
TreeMix	https://github.com/carolindahms/TreeMix	v1.13
Fastsimcoal	https://speciationgenomics.github.io/fastsimcoal2/	v2.6
CNVnator	https://github.com/abyzovlab/CNVnator	v.0.4.1
Kobas	http://bioinfo.org/kobas	v3.0
Genome analysis pipeline	https://github.com/xxiaoyull/cattle-genome-analysis	N/A

### Experimental model and study participant details

Whole-blood samples were collected from 30 cattle in Hong Kong, China. The animals sampled are part of a feral cattle population of approximately 1,000 individuals, with samples mainly collected from Sai Kung, Lantau Island, and the East and North New Territories of Hong Kong.^8^ Both male and female animals were included, and sex information was recorded for each individual (Table S1). All animal work was done in compliance with Animals (Control of Experiments) Ordinance Chapter 340, the Laws of Hong Kong (License Nos 16–47 to 50, DH/HA&P/8/2/5 Pt 5), and with City University of Hong Kong Ethical Committee approval. Animals were maintained under standard local husbandry conditions prior to sampling.

### Method details

#### DNA re-sequencing data

Genomic DNA was extracted from whole-blood samples of 30 feral cattle (see Experimental Model and Study Participant Details). DNA libraries were prepared and sequenced according to the standard protocols of BGI using a BGI-SEQ500 sequencer. Paired-end sequencing (2x150bp) was done with the target coverage of 30X. We checked the sequencing quality with FastQC v0.11.3 (https://www.bioinformatics.babraham.ac.uk/projects/fastqc/). In addition, we collected 124 WGS data from the NCBI Sequence Read Archive (SRA; https://www.ncbi.nlm.nih.gov/sra/), for 30 European taurine (Hereford, Angus, and Finncattle), 30 Northeast Asian taurine (Hanwoo and Fuzhou), 26 East Asian indicine (Weizhou and Leiqong) and 30 South Asian indicine (Sahiwal, Thawalam and Dhanni), four banteng, two of gaur, and two swamp buffalo (Table S1).

#### Variant calling

All reads were mapped to the *Bos taurus* reference genome ARS-UCD1.2 using BWA-MEM v0.7.13-r1126.^55^ SAMtools v1.9^56^ and Picard tools v2.20.2 (http://broadinstitute.github.io/picard) were used to sort the BAM file and filter duplicate reads. All genomic data were uniformly processed following the approach of a previous study.^5^ Variants were identified using ‘HaplotypeCaller’, ‘GenotypeGVCFs’, ‘SelectVariants’ and ‘VariantFiltration’ in the Genome Analysis Toolkit (GATK v3.8-1-0-gf15c1c3ef).^57^ Only biallelic alleles were retained for downstream analyses. Additionally, we performed functional annotation on all autosomes using ANNOVAR v2016-02-01.^58^

#### Comparison of findings between SNP array and WGS

Previously, the genetic composition of 21 HKF cattle was investigated with Illumina BovineSNP50v2 BeadChip.^8^ We downloaded the SNP data of these 21 animals to compare allele frequency spectrum and overlapping SNPs with our WGS data. There were only two animals in common between the two datasets. The genomic coordinates of the SNP array markers were determined according to the ARS-UCD1.2 genome assembly (liftover coordinates available at https://www.animalgenome.org/repository/cattle/UMC_bovine_coordinates/). Minor allele frequency, heterozygosity and inbreeding coefficients were computed using PLINK v1.9.^59^

#### Population structure and genetic differentiation analysis

For the population structure analysis, we filtered the SNPs with MAF < 0.01 and high linkage disequilibrium (LD) using PLINK v1.9.^59^ The LD filtering parameter was ‘--indep-pairwise 50 10 0.1’, corresponding to an r^2^ threshold of 0.1. This gave us 3,504,555 SNPs for principal component analysis (PCA) using EIGENSOFT v4.2.^60^ Using the same dataset, genetic admixture analysis was performed with ADMIXTURE v1.3.0.^61^ For the phylogenetic analysis, we selected SNPs with MAF < 0.01, resulting in 46,167,659 SNPs for tree construction. We calculated a matrix of pairwise genetic distances and constructed the neighbor-joining (NJ) phylogenetic tree with MEGA v.11.0.^62^

For 84 male samples, we obtained Y chromosomes with an average sequencing depth of 8.39x. All clean reads were aligned to the Btau_5.0.1 Y chromosome reference sequence (GCF_000003205.7), and SNPs with more than 10% missing genotypes were filtered out. A total of 2823 SNPs passed the quality control. Phylogenetic trees were then inferred using ML methods performed with MEGA v.11.0.^62^

*F*_ST_ were calculated using the ‘smartpca’ command from EIGENSOFT v4.2^60^ with parameters ‘fstonly’. The nucleotide diversity of each breed was assessed by VCFtools v0.1.16^63^ with parameters: '--window-pi 50000 --window-pi-step 20000’.

#### Runs of homozygosity

ROH were calculated using PLINK v1.9 based on phased and imputed SNPs. To characterize the distribution of ROH lengths, we applied a Gaussian mixture model implemented in the ‘mclust’ R package per population.^64^ The ROH segments were classified into short, medium and long size classes following the recommended approach.^9^^,^^65^ The boundaries of ROH were calculated to compare distribution patterns across different populations.

To evaluate specific ROH in HKF cattle, we first defined common ROH within a population as those present in more than 50% of individuals. We then used BEDTools v.2.25.0^66^ to compare ROH across multiple populations, retaining regions present in HKF but absent in other populations as specific ROH to HKF cattle.

#### Screening of loss-of-function mutations

We estimated the LoF mutations in all population genomes using SnpEff v4.3.^67^ We used the swamp buffalo genomes to infer ancestral alleles in the HKF genome to screen derived alleles. Here, we considered ‘stop_gained’, ‘stop_lost’, ‘start_lost’, ‘splice_donor_variant’, and ‘splice_acceptor_variant’ as LoF mutations. We counted the number of LoF mutations per individual in the homozygous and heterozygous states and calculated the proportion of homozygous-derived SNPs with the following formula: 2 × homozygous sites/(2 × homozygous sites + heterozygous sites).

#### Estimation of effective population size

To infer effective population sizes (Ne), we applied both MSMC2^16^ and SMC++^15^ analyses. MSMC2 was applied to all populations using two deep-coverage individuals per group, while SMC++ was computed on 15 groups of randomly selected 10 samples in the HKF population. In accordance with the recommendation, we masked low-complexity regions of the genome identified by RepeatMasker v.4.0.5 (http://www.repeatmasker.org), and focused on biallelic SNPs. For demographic inference, we used a generation time of six years and a per-generation mutation rate of 1.26 × 10^-8^.

#### Selective sweep analysis

We calculated the Di statistic^24^^,^^68^ to identify SNPs with highly differentiated allele frequencies between HKF and other cattle populations. For each SNP, Di measures the standardized deviation of pairwise *F*_ST_ values from the mean across populations, with higher values indicating greater population differentiation. We then selected the top 0.1% of Di values for further downstream functional analysis. We also used a *F*_ST_ and *θπ* ratio to detect selection sweeps within 50 kb windows and 20 kb steps in HKF cattle. The genomic selection regions considered as important were those identified using both methods.

#### ATAC-seq, ChIP-seq and transcription factor footprinting analyses

A total of ATAC-seq and ChIP-seq data from seven tissues of two animals (n=2) were obtained from NCBI. The raw reads were filtered using Trim Galore v.0.6.10 (https://github.com/FelixKrueger/TrimGalore) and mapped to the ARS-UCD1.2 reference genome using Bowtie2 v.2.4.5.^69^ Duplicates and low-quality alignments were removed with Picard v.2.20.2 (http://broadinstitute.github.io/picard). The ATAC-seq peaks were called using macs2 v.2.2.7.1.^70^ The two duplicates of the same tissue were merged using BEDTools v.2.25.0.^66^ The same pipeline was used for processing the ChIP-seq data. Transcription factor binding sites were predicted using FIMO from the MEME suite,^68^^,^^71^ with motifs obtained from the JASPAR CORE 2024 database.^72^

#### Introgression analysis

We used the *D* statistics^17^ and RFMix v2.02^18^ methods to characterize potential banteng or gaur introgression into HKF cattle. To avoid bias in the introgression ratio, we selected five pure South Asian indicine cattle (SAI), retrieved from a previous publication,^5^ for subsequent *D* statistics calculations using ADMIXTOOLS v7.0.2.^17^ RFMix v2.02^18^ was used to determine introgressed ratios based on phased data. In addition, we inferred a population-level phylogeny and migration events using qpgraph in admixtools2 (https://uqrmaie1.github.io/admixtools/articles/admixtools.html) and TreeMix.^19^ We used the “find_graphs” function to find the best-fitting admixture graph, with the swamp buffalo set as the outgroup.^73^^,^^74^ We further explored demographic models and estimated the timing of introgression based on the qpgraph results using fastsimcoal v2.6.^20^ For the demographic model, we performed 100 optimization runs with ‘-n100000’ and ‘-L20’ to obtain the best-fitting parameters yielding the highest likelihood.

To detect regions of adaptive introgression, we used U50_SAI, HKF cattle, banteng/gaur_ (1%, 50%, and 100%) based on 50 kb windows with 20 kb steps. For each window, we counted the number of SNPs where a specific allele was fixed (100% frequency) in banteng/gaur, its frequency in SAI cattle was less than 1% and greater than 50% in HKF cattle. Windows in the top 5% of such allele counts that met these criteria were considered as candidate introgressed regions.

#### Copy number variations calling and detection

The CNVnator v.0.4.1 software was used to call CNVs relative to the ARS-UCD1.2 reference assembly. The bin size was set to 250bp for each individual. All detected events had a length >1 kb, a p-value based on t-test statistics <0.001 and a fraction of reads with zero mapping quality (q0) < 0.5. CNVRs were defined using TCAG-WGS-CNV-workflow (https://github.com/bjtrost/TCAG-WGS-CNV-workflow). We considered CNVs with at least 50% reciprocal overlap as CNVRs.

To detect genetic variants involved in the coat color phenotype of HKF cattle, we calculated V_ST_ by comparing black and yellow HKF cattle. CNVRs with V_ST_ values in the top 1% of the empirical distribution were identified as putative selective sweeps associated with coat color.^75^^,^^76^

#### Annotation and functional enrichment analysis

We obtained candidate genes by annotating candidate selective sweep regions, introgressed regions and CNVRs. These candidate genes were analyzed using kobas 3.0^77^ to explain their biological functions (corrected *P* value < 0.05). The statistical significance of enriched terms was assessed using the hypergeometric test or Fisher’s exact test, and the Benjamini and Hochberg method was applied to control the false discovery rate (FDR).^78^

### Quantification and statistical analysis

All statistical details are provided in the corresponding figure legends. For comparisons of cumulative ROH length among populations, pairwise statistical analyses were performed using the Wilcoxon rank-sum test. Statistical significance is indicated by asterisks: ∗*P* < 0.05, ∗∗*P* < 0.01, ∗∗∗*P* < 0.001, and ∗∗∗∗*P* < 0.0001. Demographic models were inferred using fastsimcoal, and parameter uncertainty was assessed using 95% confidence intervals.

## Supplemental information

Document S1. Figures S1–S10

Table S1. Summary of sequencing data, related to STAR Methods

Table S2. Functional classification of the detected SNPs, related to Figure S1

Table S3. Comparison of allele frequency between SNP50 array and WGS of HKF cattle, related to STAR Methods

Table S4. The results of the 4-population test (qpDstat) in populations, related to Figure 3

Table S5. The results of the 4-population test (qpDstat) in individuals, related to Figure 3

Table S6. Results from the enrichment analysis of genes under introgressed analysis in Hong Kong feral cattle Corrected *P* value of <0.05 were considered statistically significantly enriched. Related to Figure S9

Table S7. Twelve mutations affecting ADRA1A enhancer/promoter activity, related to Figure 4

Table S8. Summary of predicted motif information, related to Figure 4

Table S9. The statistics of CNV count, related to Figure 5

Table S10. Results from the enrichment analysis of genes under CNV analysis in Hong Kong feral cattle Corrected *P* value of <0.05 were considered statistically significantly enriched. Related to Figure 5
