# Supplementary material for: The genome landscape of Hong Kong feral cattle as a unique genetic resource
Source: iScience. 2026 Mar 24;29(4):115465. doi: 10.1016/j.isci.2026.115465 (PMC13091034; doi:10.1016/j.isci.2026.115465)
Supplement: Document S1. Figures S1–S10 [file mmc1.pdf]

iScience, Volume *29*

Supplemental information

The genome landscape of Hong Kong feral cattle as a unique genetic resource

Xiaoyu Luo, Xiaoran Lu, Yan Ren, Yifan Cao, Xuewei Liu, Mario Barbato, Paolo Ajmone-Marsan, John L. Williams, Rick Tearle, Richard A.L. Brown, Michael P. Reichel, Chuzhao Lei, Ningbo Chen, and Wai Yee Low

Supplementary Information

SUPPLEMENTARY FIGURES

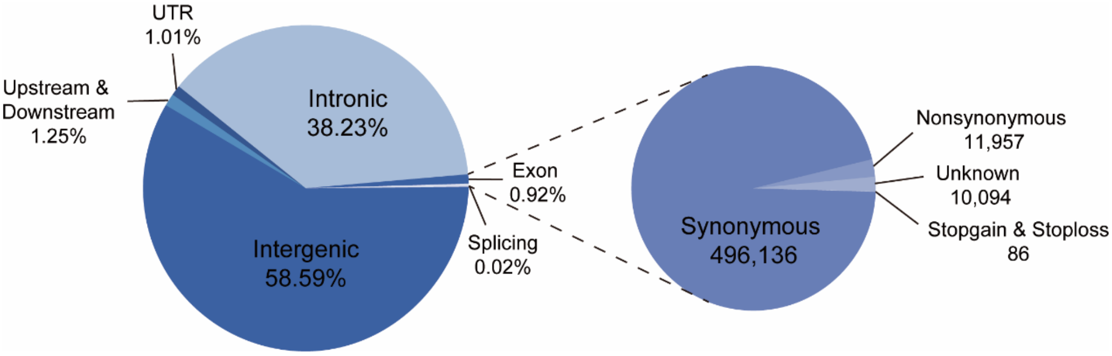

Figure S1. The functional annotation of identified SNPs. Related to Figure 1.

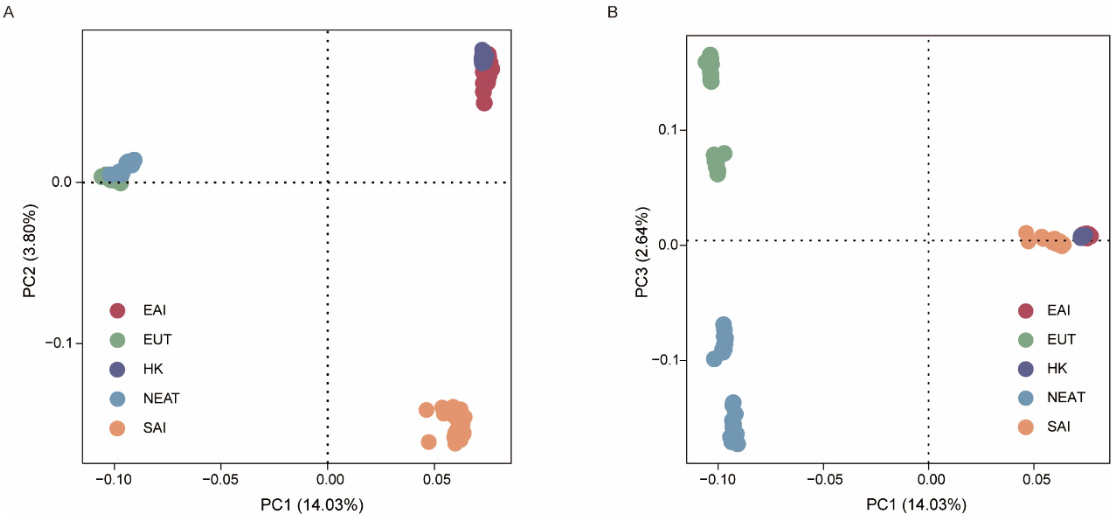

Figure S2. PCA results for 146 samples, related to Figure 1. A. PC1 and PC2 results for all samples. B. PC1 and PC3 results for all samples.

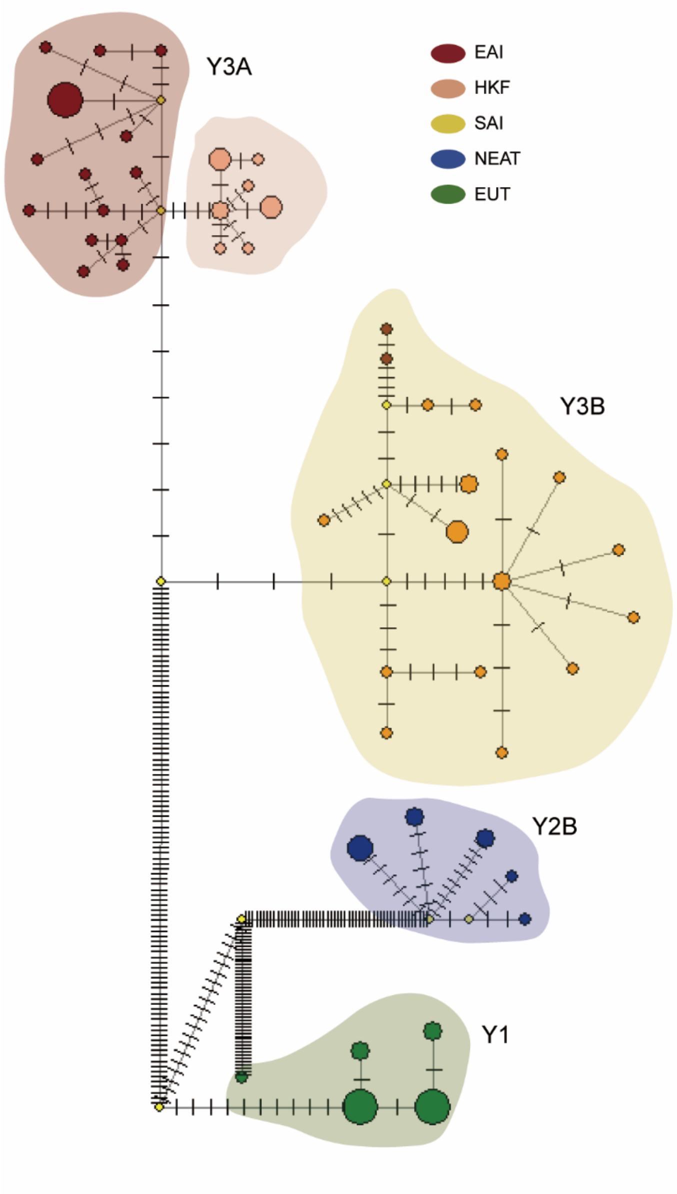

Figure S3. Phylogenetic tree of 84 Y chromosomal haplotypes in the male-specific region of the bovine Y chromosome. Related to Figure 1.

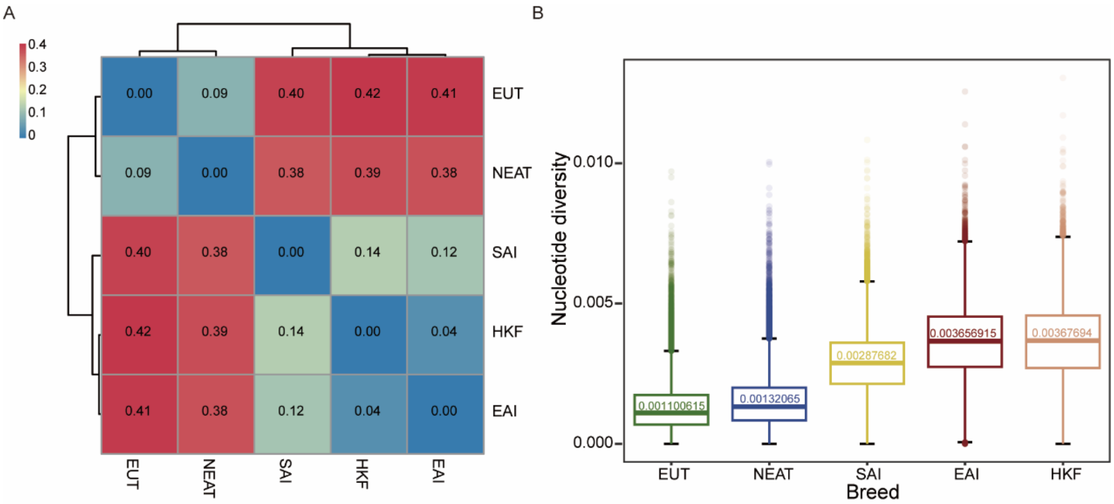

Figure S4. Analysis of genetic diversity in all populations, related to Figure 1. A. Mean pairwise F_{ST} values between all cattle populations. B. Boxplot of genome-wide nucleotide diversity between populations.

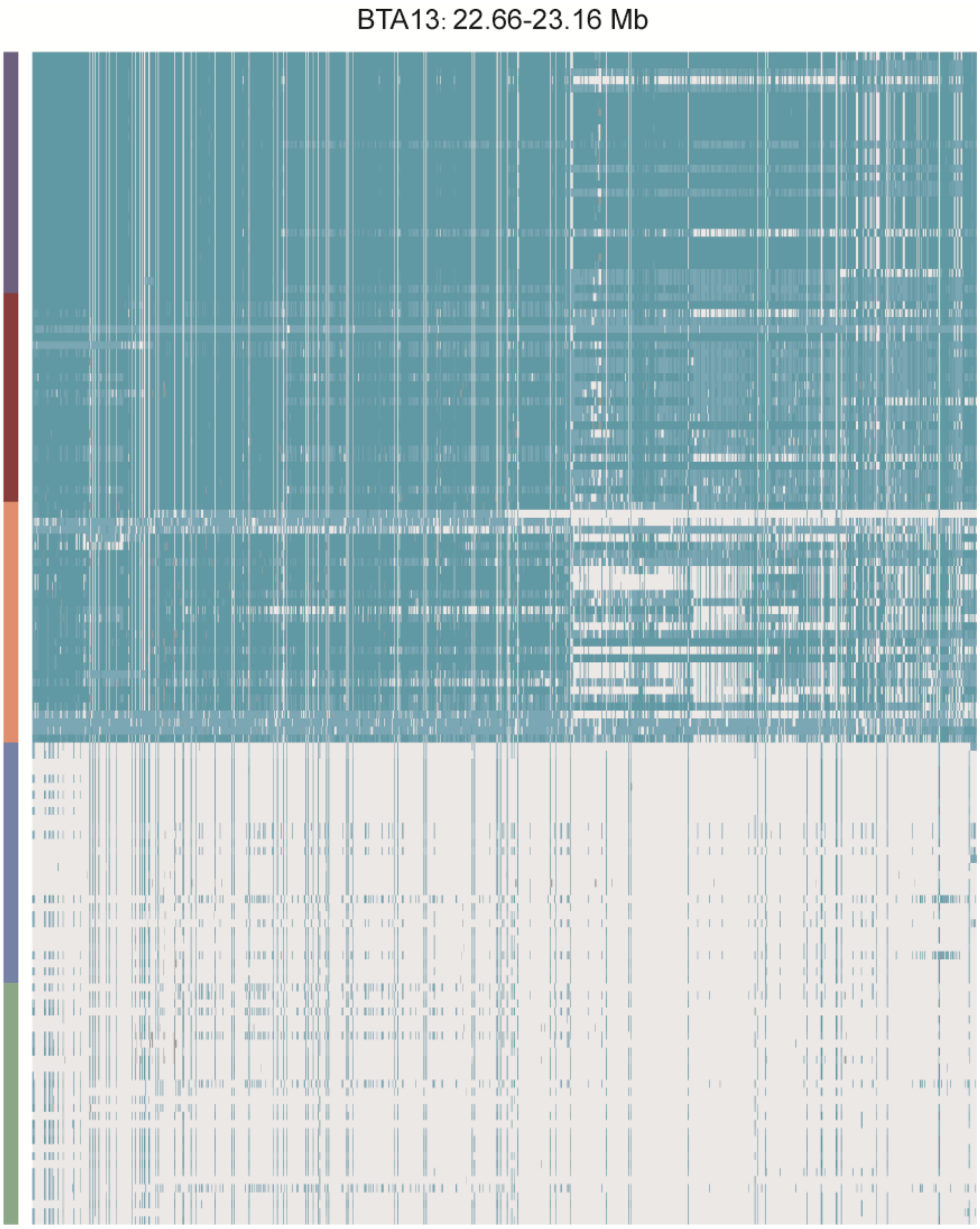

Figure S5. Haplotype analysis of the region BTA13 (22.66-23.16 Mb), which encompasses the *CASC10*, *SKIDA1*, *MLLT10*, and *DNAJC1* genes. Related to Figure 2.

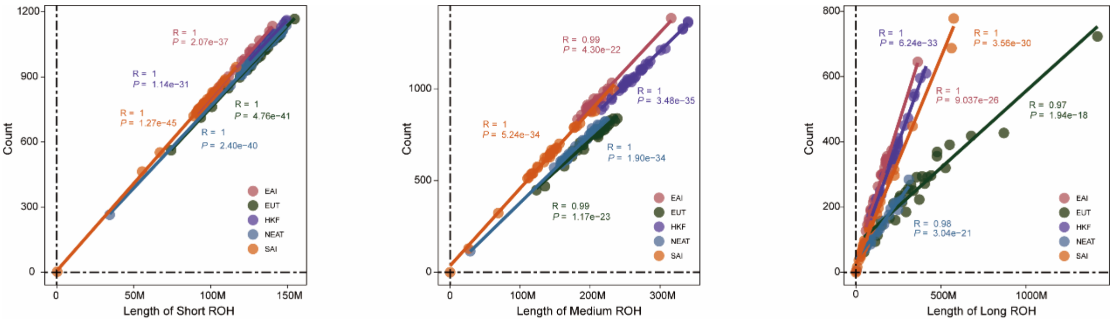

Figure S6. Cumulative length and count of ROH correlated in all populations. Related to Figure 2. A. Cumulative length and count of short ROH correlated across all cattle populations based on the Gaussian mixture model. B. Cumulative length and count of medium ROH correlated across all cattle populations based on the Gaussian mixture model. C. Cumulative length and count of long ROH correlated across all cattle populations based on the Gaussian mixture model.

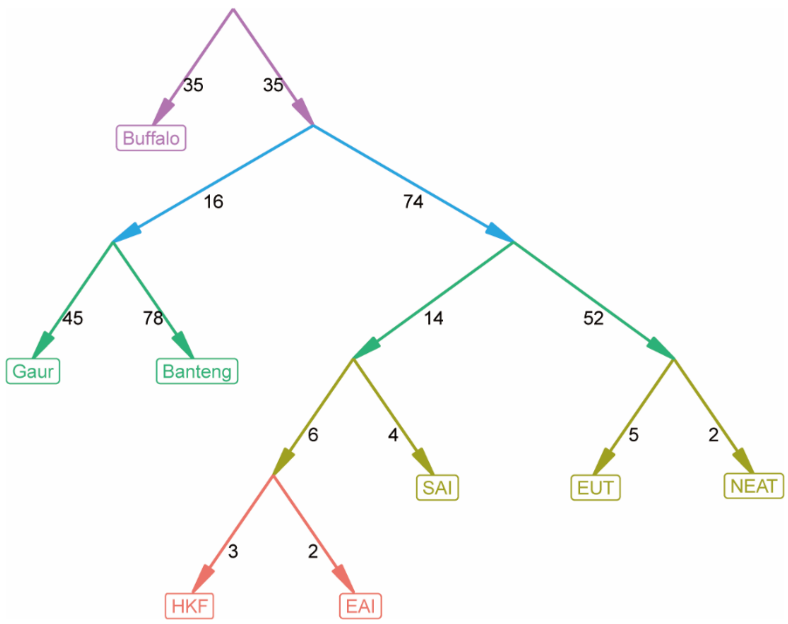

Figure S7. Origin history with no admixture events inferred by qpgraph. Related to Figure 3.

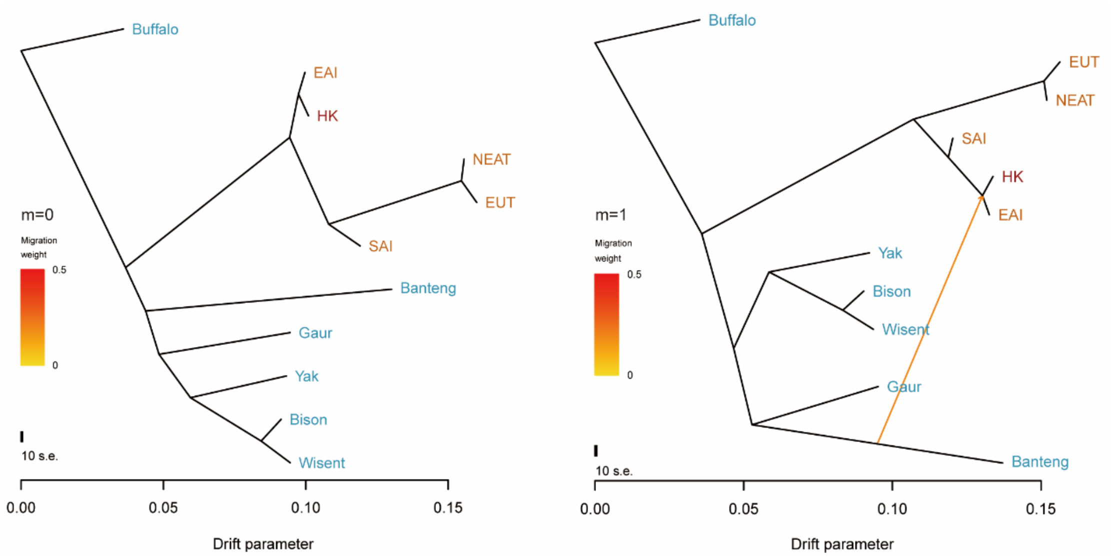

Figure S8. Inferences in population splits and admixture using TreeMix. Related to Figure 3.

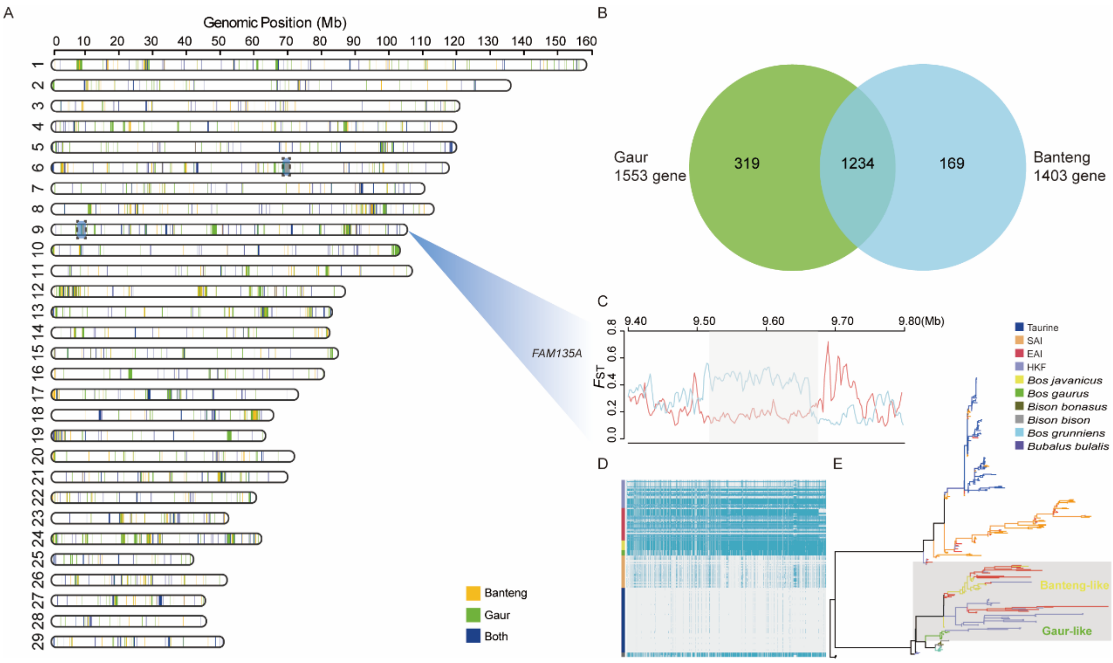

Figure S9. Introgression segments and signals impact on genomic diversity in HKF cattle. Related to STAR Methods. A. Distribution of the Introgression segments with proportions in HKF cattle according to the results of the *U50* statistic. B.Venn diagram of the number of introgressed genes from banteng or gaur into HKF cattle. C. Pairwise F_{ST} values for *FAM135A* gene. D. Haplotype patters for the candidate genes in all breeds. E: Phylogenetic trees for the candidate genes in all breeds.

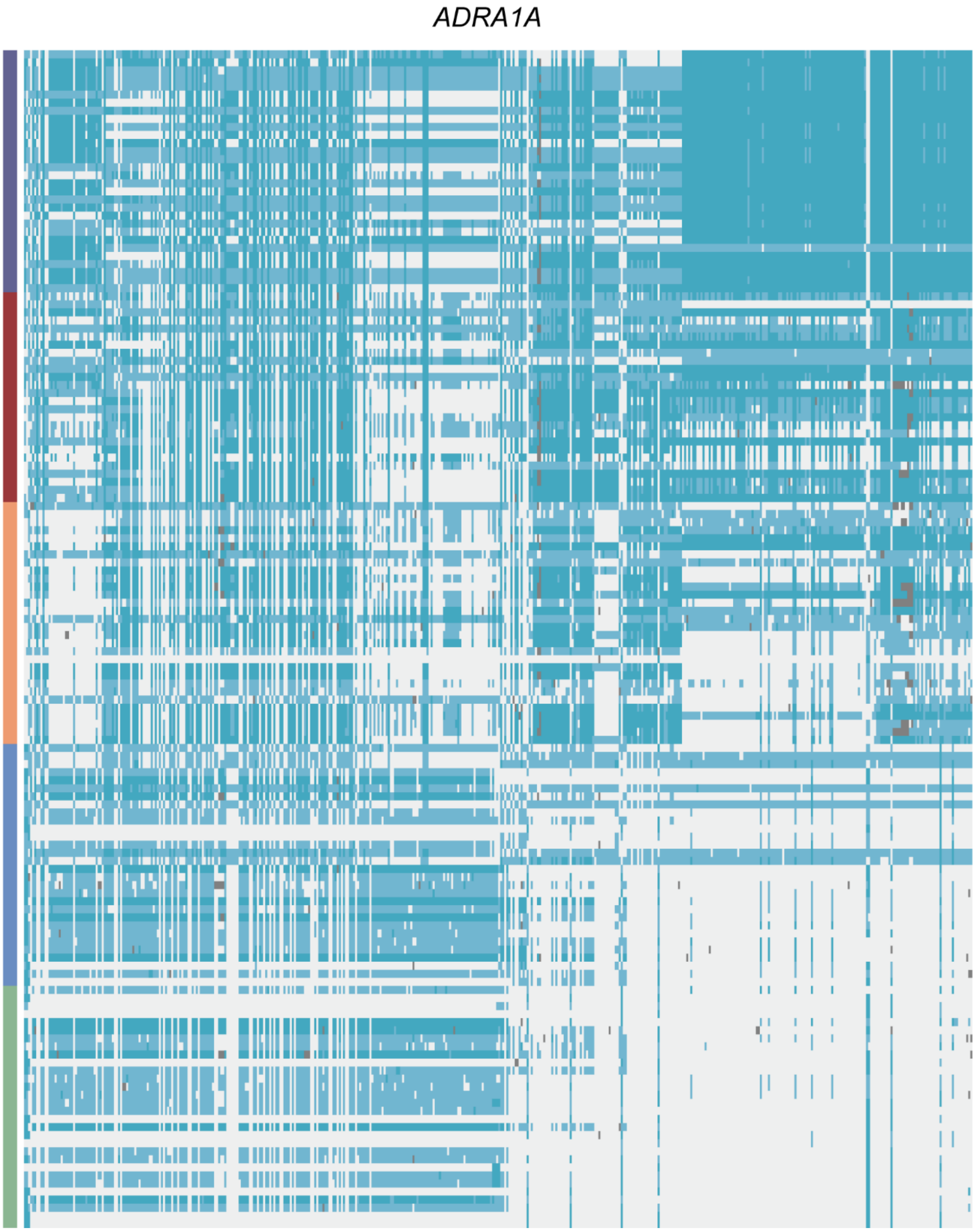

Figure S10. Distribution of *ADRA1A* haplotypes, related to Figure 4.
